# Supplementary material for: Anxiety as a mediator of relationships between perceptions of the threat of COVID-19 and coping behaviors during the onset of the pandemic in Poland
Source: PLoS One. 2020 Oct 30;15(10):e0241464. doi: 10.1371/journal.pone.0241464 (PMC7599044; doi:10.1371/journal.pone.0241464)
Supplement: Attachment — Submitted filename: Response to reviewers.docx [file pone.0241464.s002.docx]

**Response to reviewers**

PONE-D-20-10016

*Anxiety as a Mediator of Relationships between Perceptions of the Threat of COVID-19 and Coping Behaviors during the onset of the Pandemic in Poland*

6. Review Comments to the Author

Please use the space provided to explain your answers to the questions above. You may also include additional comments for the author, including concerns about dual publication, research ethics, or publication ethics. (Please upload your review as an attachment if it exceeds 20,000 characters)

Reviewer #1:

Authors have addressed points raised in the first round of reviews and the revised MS is improved as a whole. Below a few, minor, points that can improve the final MS. In the opening paragraphs of the introduction the main focus of research could be better communicated. I am referring in particular in the logical progression from the second to the third paragraph.

**Reply**: Good point. We have revised the third paragraph to make this clearer.

For the sake of parsimony, at places you could specify that the research is on negative emotion/emotional states/emotional reactions and not emotion/s at large. This can actually improve argumentation at places (i.e., p. 4 ‘More specifically, we examined if emotions mediate relationships between perceived threat and coping behavior’ and p. 5 ‘in this paper, we focus on the mediating role of emotions’ and elsewhere).

**Reply**: We have added “negative” when appropriate in numerous instances, and as noted below, we have used the term “emotional reactions.”

Similarly, you could provide examples of supporting research to statements such as in p. 3 “Our focus on relationships between threat and coping and between threat and emotions was based on previous research that has consistently found that the perceived threat of an infectious disease is positively related to engaging in coping behaviors.”

**Reply**: Good point. We added three citations for this specific point, and we included a reference in the discussion to research on self-efficacy in health behavior.

The terms emotion, emotions, emotional reactions, affect, affective reactions etc. are used interchangeably throughout the MS. This could be flagged in a footnote. In the first round of reviews I suggested to reserve the plural form of the term (i.e., emotions) to categorical forms of emotional states. Generally, the singular form tends to be used to signify more broad emotion-related processes such as the ones examined in this study. Such a stance could also ease the issue of considering (or not) Hopelessness and powerlessness (p. 22) an ‘emotion’ (I would not consider those as emotions).

**Reply**: The reviewer makes a good point. We agree that it is important to be precise, and when referring to the present study, we have used the phrase “emotional reaction,” not “emotion,” throughout the paper. PLOS One does not permit footnotes, so we have inserted a brief sentence (top of p. 4, revision) describing the fact that our use of emotional reactions is broader than the construct of emotions as used in formal models of emotions.

p. 6 "Responses to …. different perspectives". Unless you provide some examples of some of the perspectives referred to in this sentence, the second part of the sentence in particular, is quite vague in meaning.

**Reply**: We agree that this sentence is vague. It was intentionally so because as noted in the last sentence of this paragraph, we wanted to address the issue of alternative perspectives in the discussion, something we did. We did not think we needed to review in the introduction (even in passing), the sundry models that have been used to explain reactions to pandemics because our study was not designed to compare and evaluate these other approaches.

p. 12 ‘the’ WHO

**Reply**: In this instance, the article “the” is not appropriate because WHO is serving as an adjective.

p. 21 meta analysis

**Reply**: Done
